# Investigation of Physicochemical Indices and Microbial Communities in Termite Fungus-Combs

**DOI:** 10.3389/fmicb.2020.581219

**Published:** 2021-01-15

**Authors:** Guiying Yang, Farhan Ahmad, Qihuan Zhou, Meixia Guo, Shiyou Liang, Hassan Ahmed Gaal, Jianchu Mo

**Affiliations:** ^1^Ministry of Agriculture Key Laboratory of Molecular Biology of Crop Pathogens and Insect Pests, Institute of Insect Sciences, College of Agriculture and Biotechnology, Zhejiang University, Hangzhou, China; ^2^Entomology Section, Central Cotton Research Institute, Sakrand, Pakistan; ^3^Department of Entomology, Faculty of Veterinary and Animal Husbandry, Somali National University, Mogadishu, Somalia

**Keywords:** *Termitomyces*, fruiting, bacteria, fungus, cultivation, termite

## Abstract

*Termitomyces* species are wild edible mushrooms that possess high nutritional value and a wide range of medicinal properties. However, the cultivation of these mushrooms is very difficult because of their symbiotic association with termites. In this study, we aimed to examine the differences in physicochemical indices and microbial communities between combs with *Termitomyces* basidiomes (CF) and combs without *Termitomyces* basidiomes (CNF). High-performance liquid chromatography (HPLC), inductively coupled plasma optical emission spectrometry (ICP-OES), gas chromatography equipped with a flame ionization detector (GC-FID), some commercial kits, high-throughput sequencing of the 16s RNA, and internal transcribed spacer (ITS) were used. Humidity, pH, and elements, i.e., Al, Ba, Fe, Mn, Ni, S, Ca, and Mg were higher while amino acids particularly alanine, tyrosine, and isoleucine were lower in CF as compared to CNF. The average contents of fatty acids were not significantly different between the two comb categories. The bacterial genera *Alistipes*, *Burkholderia*, *Sediminibacterium*, and *Thermus* were dominant in all combs. *Brevibacterium*, *Brevundimonas*, and *Sediminibacterium* were significantly more abundant in CF. Basidiomycota and Ascomycota were also identified in combs. *Termitomyces clypeatus*, *Termitomyces* sp. Group3, and *Termitomyces* sp. were the most dominant species in combs. However, any single *Termitomyces* species was abundantly present in an individual comb.

## Introduction

*Termitomyces* is a rare genus among wild edible mushrooms, most abundantly present in (sub)tropical areas of Southeast Asia and Africa (Aanen et al., 2009; Siddiquee et al., 2015). *Termitomyces* mushrooms are widely consumed because of their taste, flavor, and medicinal benefits (Zhao et al., 2016; Hsieh and Ju, 2018). A wide spectrum of degrading enzymes is produced by *Termitomyces*, which could be used in industrial manufacturing such as papermaking (Aanen and Eggleton, 2005; da Costa et al., 2018; Banerjee et al., 2019).

*Termitomyces* appears only on the nests of fungus-growing termites (Macrotermitine) during rainy seasons. The mutualistic association between *Termitomyces* and termites started 24–34 million years ago in rainforests of sub-Saharan Africa (Aanen and Eggleton, 2005; Mueller et al., 2005; da Costa et al., 2018). Approximately 330 species of fungus-growing termites have been reported so far, which domesticate 30 described species of *Termitomyces* (Kirk et al., 2008; Makonde et al., 2013). Individual termite colonies have been found to cultivate only single species of *Termitomyces* (Makonde et al., 2013). In this symbiotic association, old termite workers forage plant materials from the outside environment and store them inside the colony (Poulsen et al., 2014). Young termite workers ingest this stored plant biomass along with *Termitomyces* nodules containing abundant asexual spores and excrete lignin-rich excrements to build fresh fungus comb (da Costa et al., 2019a). *Termitomyces* grow on the fresh comb and decompose it efficiently. After 45 days of growth, the comb is mature. Well-decomposed mature combs are consumed by the older workers (Li et al., 2017). Mature combs produce basidiomes during rainy seasons. The evolution of this intricate relationship makes it extremely difficult to cultivate *Termitomyces* mushrooms artificially, although their mycelium can grow on several nutritive media and substrates (Tibuhwa, 2012; Ono et al., 2016; da Costa et al., 2019b).

*Termitomyces* rely on some nutrients from combs to fulfill most parts of their life cycle. Some studies investigated the composition of physicochemical properties such as pH, moisture, and soluble protein in combs and termite guts (Gomathi et al., 2009; Zeleke et al., 2013). Amino acids are important nutritional sources for most fungi. They play central roles both as building blocks of proteins and as intermediates in metabolism (Wu, 2009). The fungus comb nodules are enriched with amino acids (Chiu et al., 2019). Bacterial communities in combs also play significant roles. In recent years, some researchers identified various bacteria and fungi in combs (Otani et al., 2014; Li et al., 2016; Liang et al., 2020). Some strains isolated from combs can stimulate the hyphal growth of *Termitomyces* (Sawhasan et al., 2012).

In this study, we compared physicochemical parameters including contents in amino acids, elements, sugars, fatty acids, and other basic indices between combs without *Termitomyces* basidiomes (CNF) and combs with *Termitomyces* basidiomes (CF) of the fungus-growing termite, *Odontotermes formosanus*. Microbial communities (bacteria and fungi) were also identified.

## Materials and Methods

### Collection and Preparation of Fungus Combs

Six healthy fungal combs of *O. formosanus* were collected from a forested area of Hangzhou City, Zhejiang Province, P. R. China (N 30°18′, E 120°5′). There were three CNF and three CF. Samples were wrapped in plastic bags separately and transported to the laboratory within 6 h of excavation. For each sample, the pH and moisture contents were estimated immediately, and the rest of the combs were stored at −80^°^C for subsequent experiments.

### Basic Chemical Compositions of Fungus Combs

Moisture content and pH were tested as described by Zeleke et al. (2013) with some modifications. Approximately, 1 g of each sample was ground and dissolved with 5 ml distilled water, centrifuged at 8,000 × g for 10 min; the supernatants were used for the determination of pH. Moisture content was estimated by drying the comb to a constant weight. Contents of total soluble protein, total reducing sugar, and polysaccharides including lignin, hemicellulose, and cellulose were determined by using commercial kits (Comin Biotechnology Co., Ltd., Suzhou, China).

### Element Content Determination

Inductively coupled plasma optical emission spectrometry (ICP-OES) was used to quantify elements. The procedure for determining the element contents from the samples was similar to that of Toyama-Kato et al. (2003) with minor modifications. Around 0.1 g comb was dried at 40^°^C, digested, heated to dryness, dissolved with 20 ml HNO_3_ (1 M), and filtered using a membrane filter (pore size 0.22 μm). Then, 1 ml filtered solution was diluted 10 times with 1 M HNO_3_ for the detection of calcium and magnesium separately. The operating conditions were shown in Supplementary Table S1.

### Determination of Amino Acids

The extraction procedure: Approximately 0.05 g dried comb with 5 ml HCl (6 mol/L, containing 0.1% phenol) was ground and transferred to an Eppendorf tube. After hydrolysis for 20 h at 100^°^C, 1 ml hydrolysate was collected in a petri dish and cooled down to room temperature and blown to almost dryness using a nitrogen blowing instrument (NBI, Organomation, United States). Then, 1 ml HCl (0.1 mol/L) was added to redissolve, and the turbid liquid was filtered.

Samples and standards derivatization: 200 μl comb extract from the extraction procedure mentioned above and 200 μl standard solution of 17 amino acids were transferred to Eppendorf tubes with 20 μl norleucine as an internal standard. Then, 200 μl triethylamine acetonitrile and 100 μl phenyl isothiocyanate–acetonitrile were added. After mixing, tubes were stable for 1 h at 25^°^C. Finally, 400 μl n-hexane was added in each tube, left for 10 min after shaking, and then the lower solutions were collected and diluted five times, then filtered (pore size 0.45 μm). Finally, a 10-μl sample was injected for each analysis.

The amino acid analysis was carried out with a high-performance liquid chromatography (HPLC) system (Rigol L3000, Beijing RIGOL Technology Co., Ltd., China) equipped with a UV detector (HPLC-UV), and the column was Kromasil C18 (250 mm × 4.6 mm, 5 μm). The chromatographic conditions were: a flow rate at 1 ml/min, mobile phase A was sodium acetate–acetonitrile solution and phase B was 80% acetonitrile solution. The gradient was: 100% A for 15 min, 90% A with 10% B for 10 min, 70% A with 30% B for 8 min, 55% A with 45% B for 1 s, 100% B for 5 min, and 100% A for the last 7 min.

### Volatile Fatty Acid Analysis

Different concentrations of standard solutions (10, 50, 100, 200, and 500 μg/ml) were prepared for formic acid, acetic acid, propionic acid, n-butyric acid, n-valeric acid, n-hexylic acid, and n-heptanoic acid. About 100 μg/ml 2-ethylbutyric acid solution was prepared as an internal reference solution.

Approximately 1 g sample was weighed and homogenized with 5 ml ethyl alcohol for 3 min, then kept at room temperature and shaken occasionally for 10 min. The suspension was transferred into an Eppendorf tube and centrifuged for 20 min at 6,000 rpm. The internal standard (2-ethylbutyric acid solution) was added according to Zhao et al. (2006), and the supernatant was injected into a gas chromatography system equipped with a flame ionization detector (GC-FID) for analysis.

The capillary column (30 m × 320 μm × 1.80 μm) was used, and nitrogen was applied at a flow rate of 15.0 ml/min as the carrier gas. The initial oven temperature was 100^°^C, maintained for 5 min, then raised to 200^°^C at 20^°^C/min and held for 5 min. Hydrogen, air, and nitrogen were the makeup gases at the flow rates of 35, 350, and 30 ml/min, respectively. The volume of the injected sample was 1 μl, and the run time for each solution was 15 min.

### Microbial Community Characterizations in Fungus Combs

DNA was extracted from approximately 0.5 g of each comb using the DNeasy PowerSoil kit (Qiagen, GER) for bacteria and omega M5635-02 kit (Omega, United States) for fungi separately, according to manufacturer’s instructions. The primers 341F (5′-CCTACGGGRBGCASCAG-3′) and 806R (5′-GGACTACNNGGGTATCTAAT-3′) were selected to amplify the V3-V4 variable regions of the bacterial 16S r RNA gene (Otani et al., 2014; Liang et al., 2020). ITS1 (5′-TCCGTAGGTGAACCTGCGG-3′) and ITS4 (5′-TCCTCCGCTTATTGATATGC-3′) were used to amplify the fungal ITS region (Makonde et al., 2017). The amplification reaction for bacteria was performed in 25 μl final mixture [14.75 μl dd H_2_O, 2 μl dNTP (2.5 mM), 5 μl 5 × Reaction buffer, 1 μl of each primer (10 μM), 1 μl DNA template, and 0.25 μl Fast pfu DNA Polymerase (NEB, United States)], and the conditions for PCR were 98^°^C for 5 min, followed by 25 cycles of 98°C for 30 s, 52°C for 30 s, and 72°C for 45 s, with a final extension at 72°C for 5 min. The fungal amplification reaction was the same as that for bacteria, and the conditions for PCR were 98^°^C for 5 min, followed by 28 cycles of 98°C for 30 s, 52°C for 45 s, and 72°C for 45 s, with a final extension at 72°C for 5 min. The final target PCR products were visualized with agarose gel electrophoresis, then extracted and purified from the gel. DNA concentrations were quantified using Quant-iT PicoGreen ds DNA Assay Kit (Invitrogen, CA) and Microplate reader (BioTek, United States). Finally, samples were sequenced commercially (Personalbio Biotech, Shanghai, China) on Illumina MiSeq platform (paired-end) for bacteria and Pacbio Sequel platform (single-molecule real-time) for fungus.

### Statistical Analysis

Differences in physicochemical parameters were tested with one-way ANOVA analysis and Student’s *t*-test using SPSS, v.20.0 at alpha = 0.05. Pyrotag reads were denoised using the DADA2 method according to Benjamini and Hochberg (1995). Raw sequences were quality-filtered according to Koljalg et al. (2013) using the QIIME Release 8.0 software. Silva (Release132)^1^ with 16S r RNA gene sequences of cockroach (Quast et al., 2013) and UNITE (Release 8.0)^2^ (Koljalg et al., 2013) were used as reference databases for bacteria and fungi, respectively. Packages (stat, ape, Venn diagram, pheatmap, and ggplot2) of R software^3^ were used to map and compare the relative abundance of bacteria and fungi at different levels. QIIME2 (2019.4) was used to calculate every index of alpha diversity including Chao1 (Chao, 1984), Observed species, Shannon (Shannon, 1948), Simpson (Simpson, 1949), Pielou’s evenness (Pielou, 1966), and Good’s coverage (Good, 1958). Kruskal–Wallis test (*H*-test) and Dunn test were applied to test for differences in alpha diversity.

## Results

### Basic Parameters of Combs

Lignin was significantly (*P* < 0.01) lower in CF (229.84 ± 25.12) as compared to CNF (277.67 ± 45.82), whereas pH (*P* < 0.05) and moisture (*P* < 0.01) were relatively higher in CF as compared to CNF. The contents in total soluble protein, total reducing sugar, hemicellulose, and cellulose were not significantly different between CF and CNF (*P* > 0.05; Table 1).

**TABLE 1:** Contents of some basic parameters.

**Parameters**	**CNF**	**CF**	**Significance**
Moisture (%)	41.37 ± 0.01	49.87 ± 0.00	0.006
pH	4.29 ± 0.01	5.00 ± 0.11	0.024
Total soluble protein (mg/g)	26.63 ± 5.84	17.33 ± 0.62	0.252
Total reducing sugar (mg/g)	3.15 ± 0.20	2.40 ± 0.25	0.081
Cellulose (mg/g)	133.47 ± 1.38	169.73 ± 3.52	0.425
Hemicellulose (mg/g)	129.46 ± 0.73	129.40 ± 0.22	0.942
Lignin (mg/g)	277.67 ± 45.82	229.84 ± 25.12	0.004

*CF, combs with Termitomyces basidiomes; CNF, combs without Termitomyces basidiomes.*

### Concentration of Elements in Combs

Eighteen elements in each sample were determined by ICP-OES. The average concentration of each element was different between CF and CNF. All elements (except Cd, Co, Na, P, and Pb) were higher in CF as compared to CNF (Table 2). Cd and Pb were lower in CF than in CNF.

**TABLE 2:** Element analysis in CNF and CF.

**Elements**	**CNF (ppm)**	**CF (ppm)**	**Significance**
Al	3.83 ± 0.41	8.28 ± 0.27	0.002
Ba	0.06 ± 0.01	0.41 ± 0.05	0.002
Cd	0.02 ± 0.00	0.00 ± 0.00	0.045
Co	0.03 ± 0.03	0.03 ± 0.01	0.935
Cr	0.01 ± 0.00	0.11 ± 0.07	0.199
Cu	0.15 ± 0.02	0.26 ± 0.05	0.155
Fe	3.71 ± 0.26	17.51 ± 1.10	0.004
K	1.72 ± 0.30	2.61 ± 0.88	0.423
Mn	1.76 ± 0.33	6.62 ± 1.48	0.033
Mo	0.03 ± 0.01	0.08 ± 0.05	0.383
Na	0.87 ± 0.01	0.87 ± 0.22	0.978
Ni	0.00 ± 0.00	0.12 ± 0.02	0.008
P	5.59 ± 0.41	4.73 ± 0.31	0.171
Pb	0.37 ± 0.01	0.04 ± 0.02	0.002
S	7.91 ± 0.43	14.84 ± 1.02	0.011
Zn	0.17 ± 0.04	0.30 ± 0.03	0.065
Ca	8.51 ± 0.37	16.05 ± 1.75	0.045
Mg	0.44 ± 0.06	0.83 ± 0.06	0.011
df	17	17	
F	12.67	12.67	
*P*-value	*P* < 0.05	*P* < 0.05	

*CF, combs with Termitomyces basidiomes; CNF, combs without Termitomyces basidiomes.*

### Amino Acid Analysis

**FIGURE 1:**
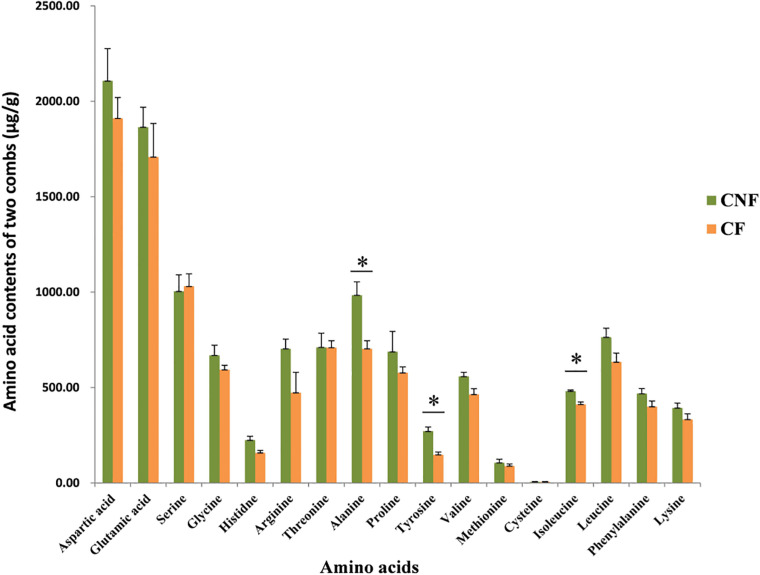
Comparisons of amino acids between combs with *Termitomyces* basidiomes and combs without *Termitomyces* basidiomes. ^∗^ indicates high significance (*P* < 0.05).

We compared 17 amino acids between CF and CNF by HPLC-UV (Supplementary Figure S1 and Supplementary Table S2). The amino acid contents were higher in CNF as compared to CF (Figure 1). Among all amino acids, aspartic acid content was highest in both comb types, i.e., CNF (2,107.24 μg/g ± 169.44) and CF (1,910.73 μg/g ± 109.67), followed by glutamic acid (1,864.38 μg/g ± 104.48 and 1,708.38 μg/g ± 175.92, respectively), while average cysteine was lowest in both combs (5.70 μg/g ± 1.93 and 6.05 μg/g ± 1.63, respectively). Amino acid profiles of combs were changed a lot after basidiome formation. Serine and cysteine contents were more abundant in CF (1,030.54 μg/g ± 65.32 and 6.05 μg/g ± 1.63, respectively) than those in CNF (1,004.41 μg/g ± 85.94 and 5.70 μg/g ± 1.93, respectively), while other amino acids were lower in CF. Surprisingly, the relative abundance of alanine, tyrosine, and isoleucine significantly decreased with the appearance of basidiomes (Student’s *t*-test, *P* < 0.05).

### Fatty Acid Analysis

**FIGURE 2:**
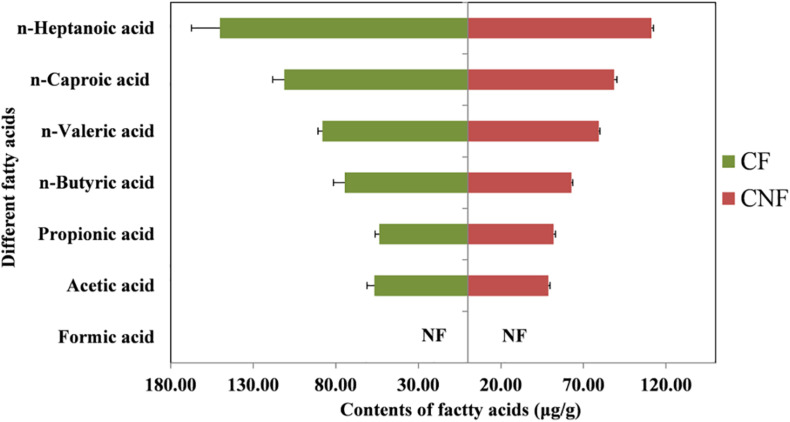
Contents of different fatty acids in combs. NF means not found.

The contents of six fatty acids were measured in CNF and CF (Supplementary Tables S3, S4). The average contents of fatty acids were not significantly different between comb categories. The average contents of caproic acid in both CNF (64.54 μg/g ± 1.20) and CF (94.59 μg/g ± 11.39) were the highest followed by butyric acid (57.80 μg/g ± 0.92 and 74.78 μg/g ± 7.93, respectively) and acetic acid (47.89 μg/g ± 1.21 and 53.86 μg/g ± 4.11, respectively). Whereas the average contents of propionic acid were lowest in CNF (1.09 μg/g ± 0.01) and CF (1.00 μg/g ± 0.04) followed by valeric acid (1.37 μg/g ± 0.02 and 1.20 μg/g ± 0.12, respectively) and heptanoic acid (1.73 μg/g ± 0.05 and 1.63 μg/g ± 0.22, respectively). We did not find formic acid in combs (Figure 2 and Supplementary Table S4).

### Bacterial Community Analysis

**FIGURE 3:**
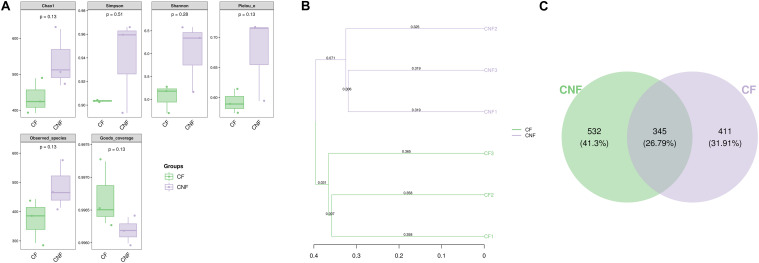
**(A)** The box plots demonstrated significance among six combs in different indicators, including numbers of sequences, coverage, richness, diversity, and evenness. **(B)** Clustering tree using unweighted pair-group method with arithmetic means (UPGMA) revealed the bacterial similarities among combs. **(C)** Venn diagram of OTU between combs without *Termitomyces* basidiomes (CNF) and combs with *Termitomyces* basidiomes (CF).

Between 21,566 and 24,198 high-quality sequences were obtained from each comb. The Good’s coverage for every comb was more than 99.6% (Supplementary Table S5). The microbial diversity indices (Shannon and Simpson), estimated community richness (Chao1 and Observed species), and evenness (Pielou’s evenness) were not statistically different between CF and CNF (Student’s *t*-test; *P* > 0.05; Figure 3A). The unweighted pair-group method with arithmetic means (UPGMA) clustering analysis grouped all combs into two distinct groups, i.e., CF and CNF (Figure 3B). The Venn diagram showed that CNF and CF occupied 532 (41.30%) and 441 (31.91%) operational taxonomic units (OTUs), respectively. The OTU 345 (26.79%) was common between both comb categories (Figure 3C).

**FIGURE 4:**
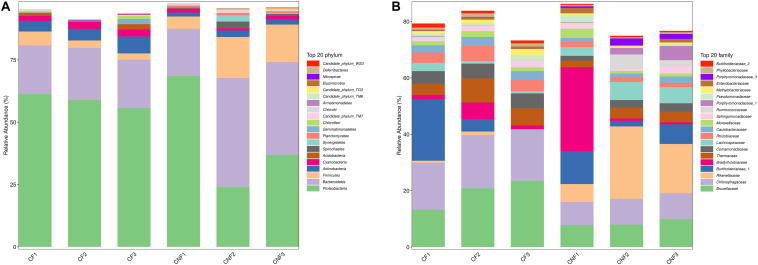
The bacterial community structure in combs with *Termitomyces* basidiomes (CF) and combs without *Termitomyces* basidiomes (CNF) combs. **(A)** The relative abundance of bacteria at the phyla level. **(B)** The bacterial composition at the family level. CF1, CF2, and CF3 represent three combs with basidiomes, whereas CNF1, CNF2, and CNF3 represent three combs without basidiomes.

The abundance of various bacterial groups at different taxonomic levels (phylum, family, and genus) was investigated. A total of 20 bacterial phyla were found and compared (Figure 4A) among all combs. Proteobacteria (23.97–68.52%), Bacteroidetes (18.85–43.83%), and Firmicutes (2.45–16.44%) were the dominant identified OTUs among all. With the growth of basidiomes, Actinobacteria and Cyanobacteria increased significantly, while Planctomycetes and Chlorobi decreased (Supplementary Table S6).

At the family level, Brucellaceae (7.82–23.56%), Chitinophagaceae (8.20–18.85%), Rikenellaceae (0.02–25.84%), Burkholderiaceae_1 (0.06–21.96%), and Bradyrhizobiaceae (0.71–29.78%) were dominant in all combs (Figure 4B). Furthermore, Chitinophagaceae (Bacteroidetes), Comamonadaceae (Proteobacteria), Rhizobiaceae (Proteobacteria), Caulobacteraceae (Proteobacteria), and Phyllobacteriaceae (Proteobacteria) were significantly high in CF (Student’s *t*-test; *P* < 0.05) (Supplementary Table S7).

**FIGURE 5:**
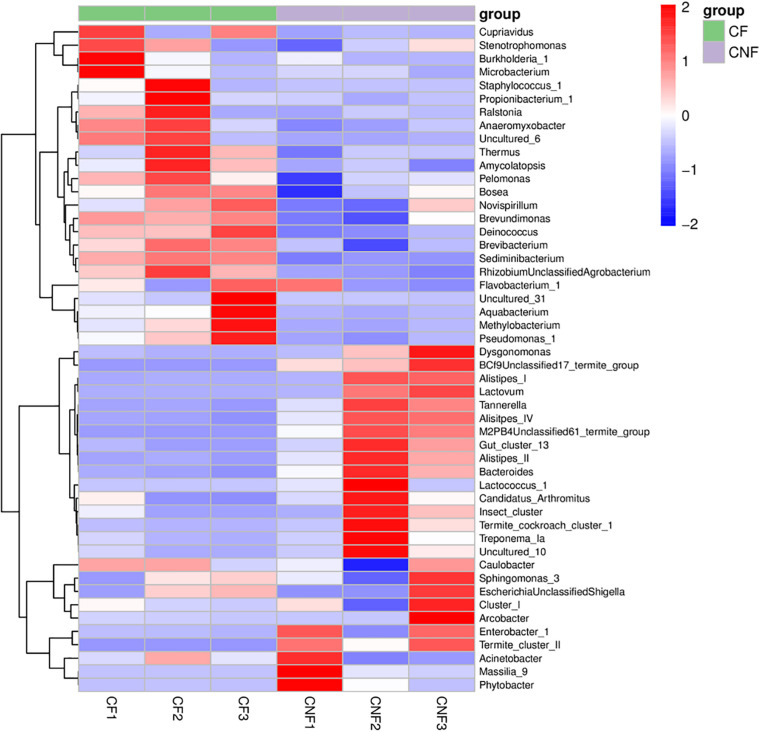
The heat map illustrated the relative abundances of bacteria among combs at the genus level.

At the genus level, *Sediminibacterium* (8.18–18.80%), *Alistipes_II* (0.02–18.99%), *Burkholderia_1* (0.01–21.40%), and *Thermus* (2.35–8.53%) were the most abundant bacterial genera in all combs. *Sediminibacterium* (Chitinophagaceae, Bacteroidetes), *Brevundimonas* (Caulobacteraceae, Proteobacteria), and *Brevibacterium* (Brevibacteriaceae, Actinobacteria) were significantly more abundant in combs with basidiomes, while the members of *Alistipes IV* (Rikenellaceae, Bacteroidetes) were significantly less abundant (Student’s *t*-test; *P* < 0.05) (Figure 5 and Supplementary Table S8).

### Fungal Community Analysis

Between 4,008 and 5,854 high-quality sequences were obtained from every comb. The coverage for every comb was closed to 100% (Supplementary Table S9). Alpha diversity indices (Shannon, Simpson, Chao1, Observed species, and Pielou’s evenness) were not significantly different between comb types (Student’s *t*-test; *P* > 0.05).

Two phyla, i.e., Basidiomycota and Ascomycota, were found in both types of combs. Basidiomycota was the most prevalent phylum. At the family level, Lycoperdaceae (the family to which *Termitomyces* belongs), Cystostereaceae, and Xylariaceae were identified. Lycoperdaceae was the most abundant in all combs. The species including *Xylaria grammica* (0.00–0.18%), *Cystidiodontia laminifera* (0.00–1.16%), *Lycoperdaceae* sp. TU112130 (0.00–2.55%), *Termitomyces clypeatus* (0.50–99.92%), *Termitomyces* sp. (0.00–2.68%), *Termitomyces* sp. Group3 (0.00–97.14%), and *fungal* sp. (0.00–1.02%) were identified. Individual combs were dominated by a single *Termitomyces* species (Table 3).

**TABLE 3:** Comparisons of fungus between CF and CNF.

**Phylum**	**Family**	**Species**	**CNF (%)**				**CF (%)**				**Sig**
			**CNF1**	**CNF2**	**CNF3**	**CNF average**	**CF1**	**CF2**	**CF3**	**CF average**	
Basidiomycota	Lycoperdaceae	*Termitomyces clypeatus*	0.50	99.92	97.32	65.91	99.92	97.45	99.89	99.09	0.368
		*Termitomyces* sp. Group3	97.14	0.00	0.00	32.38	0.08	0.00	0.00	0.03	0.374
		*Termitomyces* sp.	0.00	0.00	2.68	0.89	0.00	0.00	0.00	0.00	0.374
		*Lycoperdaceae* sp. TU112130	0.00	0.08	0.00	0.03	0.00	2.55	0.03	0.86	0.380
	Cystidiodontia	*Cystidiodontia laminifera*	1.16	0.00	0.00	0.39	0.00	0.00	0.00	0.00	0.374
Ascomycota	Xylariaceae	*Xylaria grammica*	0.18	0.00	0.00	0.06	0.00	0.00	0.08	0.03	0.649

*CF, combs with Termitomyces basidiomes; CNF, combs without Termitomyces basidiomes.*

## Discussion

The present study showed differences in physicochemical properties and microbial communities between CF and CNF. Most fungi grow well at acidic pH and low humidity (Hesse, 1955; Gomathi et al., 2009), and we indeed found low moisture and pH in all combs (Table 1). However, we disagree with the previous study reporting that moisture content decreased after basidiome formation (Sidde Gowda and Rajagopal, 1990). Less lignin was recorded from combs with basidiomes as compared to combs without basidiomes (Table 1), indicating that *Termitomyces* degrades lignin during the comb maturation period (Hyodo et al., 2000; Bashir et al., 2015).

Our results showed the variation tendency of 18 elements including 16 metal and two non-metal elements in combs (Table 2). Li et al. (2012) also found metal ions in fungus combs. Element contents were relatively higher in CF as compared to CNF. Mushrooms can absorb large amounts of water and minerals from their substrates (Koutrotsios et al., 2020). The bioaccumulation capacity of mushroom is influenced by the substrate, fungal lifestyle, age of the fruiting body, species, type of element, and environment (Campos et al., 2009; Lee et al., 2009; Koutrotsios et al., 2020). We assume that the high concentrations of elements in combs are associated with lignocellulose degradation and fungal growth. Some studies demonstrated that elements are important for fungal metabolic functions related to enzyme production and lignocellulose degradation (Li et al., 2012; Koutrotsios et al., 2020), but less information is available on the significant roles of elements in fungal growth. Cuero et al. (2003) reported that various metal ions caused some phenotypic or genotypic influences in fungus and could stimulate fungal growth. Moreover, some elements play a crucial role in enzymatic reactions regarding nitrogen fixation (Hoffman et al., 2014). For example, molybdenum, iron, and magnesium are essential for the structure formation of metal clusters in nitrogenase and MgATP energy supply (Hoffman et al., 2014), which may strengthen nitrogen fixation.

Amino acids were higher in CNF as compared to CF. This may be due to the uptake of amino acids in basidiomes from combs. It has been reported that amino acids are transported into a fungal cell from the substrate (Whitaker, 1976). *Termitomyces* mushrooms are capable of transforming amino acids or synthesizing amino acids from non-protein nitrogen (Botha and Eicker, 1992; Deacon, 2006). In termite–fungus association, the amino acids are accumulated in matured fungus combs and fungal nodules (Botha and Eicker, 1992). Chiu et al. (2019) compared 18 amino acids between fungus combs and fungal nodules of *Termitomyces*. They allocated 7% amino acids to nodules and 93% to combs. Moreover, it has also been reported that amino acids are important for fungal growth and reproduction (Steinberg, 1950; Yang et al., 2020). However, knowledge on the role of amino acids on fungal growth is less satisfactory.

All fatty acids were found in our experiment except formic acid (Figure 2 and Supplementary Table S4). Some short-chain fatty acids such as acetic acid, propionic acid, and butyric acid were also reported previously both in termite guts and fungus combs (Li et al., 2016). It has been reported that fatty acids are produced by microorganisms living in termite guts during the fermentation of fiber and starch (Odelson and Breznak, 1983; Thorburn et al., 2014; Ciarlo et al., 2016).

The bacterial communities in fungus combs are involved in lignocellulose degradation, nutrient supplementation, nitrogen fixation, and antibiotic production (Liang et al., 2020), but whether these bacteria play significant roles in the appearance of basidiomes from the comb remains to be proven. We found that the major bacterial lineages were similar among all combs, but there were marked changes in their abundance (Figures 4, 5 and Supplementary Tables S6–S8). Actinobacteria, Bacteroidetes, Cyanobacteria, Firmicutes, and Proteobacteria were plentifully found in all combs (Figure 3A and Supplementary Table S6). We are consistent with the previous studies on fungus combs (Otani et al., 2014; Liang et al., 2020). It has been reported that some members of Bacteroidetes and Firmicutes can play important roles in the metabolism of carbohydrates (Poulsen et al., 2014; Delmont et al., 2018). Proteobacteria along with Bacteroidetes and Firmicutes are related with nitrogen input, which may help meet the nutritional needs of *Termitomyces* (Poulsen et al., 2014; Delmont et al., 2018). The nitrogenase reductase *nifH* genes have been identified in Proteobacteria (Delmont et al., 2018). Liang et al. (2020) suggested that Proteobacteria in fungus comb assist fungus in atmospheric nitrogen fixation. Our findings also indicated that Cyanobacteria and Actinobacteria were more abundant in CF than in CNF (Figure 3A and Supplementary Table S6). Biologists suggested that many species of Cyanobacteria found in fungus combs can fix N_2_ (Stewart, 1980; Makonde et al., 2015). It has been described that the C/N ratio can maximize fungal growth performance *in vitro* (Shik et al., 2016). Actinobacteria have also been isolated from fungus combs previously (Visser et al., 2012; Benndorf et al., 2018; Yin et al., 2019). They are generally known as defensive symbionts because they produce antibiotic compounds to suppress invading pathogens (Visser et al., 2012; Benndorf et al., 2018; Yin et al., 2019). Based on these results, we believed that some bacteria from combs are important to create a suitable growth condition for *Termitomyces* by maintaining the C/N balance or inhibiting the infectious microbes.

Fungal communities of Basidiomycota and Ascomycota were also identified in combs; however, the members of *Termitomyces* were dominant in all combs (Table 3). These results were the same as reported previously by Shinzato et al. (2005) that *Termitomyces* was the only visible fungus on the nests of termites, and the individual nest has been found to contain only single species of *Termitomyces* within the fungus garden.

## Conclusion

The current study investigated the differences in physicochemical factors (pH, moisture, total soluble protein, total reducing sugar, cellulose, hemicellulose, lignin, amino acids, metal ions, and fatty acids) and microbial communities (bacteria and fungi) between CF and CNF. A noticeable increase of humidity, pH, and elements (Al, Ba, Fe, Mn, Ni, S, Ca, and Mg) and a decrease of some amino acids (alanine, tyrosine, and isoleucine) were recorded in CF as compared to CNF. This study also revealed a high level of bacterial diversity in fungal combs. The majority of the bacterial communities might assist *Termitomyces* in lignocellulose degradation and maintenance of the C/N ratio. The possible role of physicochemical characteristics and bacterial communities on the onset of basidiome development by *Termitomyces* remains unknown, and further in-depth studies are needed.

## Data Availability Statement

The datasets were submitted to the Sequence Read Archive of NCBI (http://www.ncbi.nlm.nih.gov; BioProject PRJNA66 2821).

## Author Contributions

GY and JM: conceptualization, project administration, and funding acquisition. GY: methodology. GY and FA: software, writing—original draft preparation, and visualization. GY, QZ, and MG: validation. GY and SL: formal analysis and data curation. GY and MG: investigation. GY and QZ: resources. FA, HG, and JM: writing—review and editing. JM: supervision. All authors have read and agreed to the published version of the manuscript.

## Conflict of Interest

The authors declare that the research was conducted in the absence of any commercial or financial relationships that could be construed as a potential conflict of interest.

## Footnotes

1 http://www.arb-silva.de

2 https://unite.ut.ee/

3 http://www.r-project.org

## Abbreviations

CF: combs with *Termitomyces* basidiomes
CNF: combs without *Termitomyces* basidiomes
HPLC: high-performance liquid chromatography
ICP-OES: inductively coupled plasma optical emission spectrometry
GC-FID: gas chromatography equipped with a flame ionization detector.

## References

[B1] Aanen D. K. de Fine Lichat H. H. Debets Alfons J. M. Kerstes Niels A. G. Hoekstra R. F. Boomsma J. J. (2009). High symbiont relatedness stabilizes mutualistic cooperation in fungus growing termites. *Science* 326 1103–1105. 10.1126/science.1173462 19965427

[B2] Aanen D. K. Eggleton P. (2005). Fungus-growing termites originated in African rain forest. *Curr. Biol.* 15 851–855. 10.1016/j.cub.2005.03.043 15886104

[B3] Banerjee S. Roy A. Madhusudhan M. S. Bairagya H. R. Roy A. (2019). Structural insights of a cellobiose dehydrogenase enzyme from the basidiomycetes fungus *Termitomyces clypeatus*. *Comput. Biol. Chem.* 82 65–73. 10.1016/j.compbiolchem.2019.05.013 31272063

[B4] Bashir H. Gangwar R. Mishra S. (2015). Differential production of lignocellulolytic enzymes by a white rot fungus *Termitomyces* sp. OE147 on cellulose and lactose. *Biochim. Biophys. Acta* 1854 1290–1299. 10.1016/j.bbapap.2015.07.005 26164778

[B5] Benjamini Y. Hochberg Y. (1995). Controlling the false discovery rate: a practical and powerful approach to multiple testing. *J. R. Statist. Soc. B* 57 289–300. 10.1111/j.2517-6161.1995.tb02031.x

[B6] Benndorf R. Guo H. Sommerwerk E. Weigel C. Garcia-Altares M. Martin K. (2018). Natural products from actinobacteria associated with fungus-growing termites. *Antibiotics* 7:83. 10.3390/antibiotics7030083 30217010 PMC6165096

[B7] Botha W. J. Eicker A. (1992). Nutritional value of *Termitomyces* mycelial protein and growth of mycelium on natural substrates. *Mycol. Res.* 96 350–354. 10.1016/S0953-7562(09)80949-0

[B8] Campos J. A. Tejera N. A. Sanchez C. J. (2009). Substrate role in the accumulation of heavy metals in sporocarps of wild fungi. *Biometals* 22 835–841. 10.1007/s10534-009-9230-7 19333556

[B9] Chao A. (1984). Nonparametric estimation of the number of classes in a population. *Scand. J. Statist.* 11 265–270.

[B10] Chiu C. I. Ou J. H. Chen C.-Y. Li H.-F. (2019). Fungal nutrition allocation enhances mutualism with fungus-growing termite. *Fungal Ecol.* 41 92–100. 10.1016/j.funeco.2019.04.001

[B11] Ciarlo E. Heinonen T. Herderschee J. Fenwick C. Mombelli M. Roy D. L. (2016). Impact of the microbial derived short chain fatty acid propionate on host susceptibility to bacterial and fungal infections in vivo. *Sci. Rep.* 6:37944. 10.1038/srep37944 27897220 PMC5126587

[B12] Cuero R. Ouellet T. Yu J. Mogongwa N. (2003). Metal ion enhancement of fungal growth, gene expression and aflatoxin synthesis in *Aspergillus flavus*: RT-PCR characterization. *J. Appl. Microbiol.* 94 953–961. 10.1046/j.1365-2672.2003.01870.x 12752802

[B13] da Costa R. R. Hu H. Pilgaard B. Vreeburg Sabine M. N. Schuckel J. Pedersen Kristine S. K. (2018). Enzyme activities at different stages of plant biomass decomposition in three species of fungus-growing termites. *Appl. Environ. Microbiol.* 84:e01815-17. 10.1128/AEM.01815-17 29269491 PMC5812949

[B14] da Costa R. R. Hu H. Li H. Poulsen M. (2019a). Symbiotic plant biomass decomposition in fungus-growing termites. *Insects* 10:87. 10.3390/insects10040087 30925664 PMC6523192

[B15] da Costa R. R. Vreeburg Sabine M. E. Shik J. Z. Aanen D. K. Poulsen M. (2019b). Can interaction specificity in the fungus-farming termite symbiosis be explained by nutritional requirements of the fungal crop? *Fungal Ecol.* 38 54–61. 10.1016/j.funeco.2018.08.009

[B16] Deacon J. W. (2006). *Fungal Biology*, 4rd Edn Oxford: Blackwell Publishing.

[B17] Delmont T. O. Quince C. Shaiber A. Esen O. C. Lee S. T. Rappe M. S. (2018). Nitrogen-fixing populations of Planctomycetes and *Proteobacteria* are abundant in surface ocean metagenomes. *Nat. Microbiol.* 3 804–813. 10.1038/s41564-018-0176-9 29891866 PMC6792437

[B18] Gomathi V. Esakkiammal M. Thilagavathi S. S. Ramalakshmi A. (2009). Environmental influence on physico-chemical and biological activities of fungus growing termite (*Isoptera*: *Macrotermitinae*). *Asia J. Bio Sci.* 4 88–92.

[B19] Good I. J. (1958). The population frequency of species and theestimation of the population parameters. *Biometrics* 40 237–246. 10.2307/2333344

[B20] Hesse P. R. (1955). A chemical and physical study of the soils of termite mounds in East Africa. *J. Ecol.* 43 449–461.

[B21] Hoffman B. M. Lukoyanov D. Yang Z. Y. Dean D. R. Seefeldt L. C. (2014). Mechanism of nitrogen fixation by nitrogenase: the next stage. *Chem. Rev.* 114 4041–4062. 10.1021/cr400641x 24467365 PMC4012840

[B22] Hsieh H. M. Ju Y.-M. (2018). Medicinal components in *Termitomyces* mushrooms. *Appl. Microbiol. Biotechnol.* 102 4987–4994. 10.1007/s00253-018-8991-8 29704040

[B23] Hyodo F. Inoue T. Azuma J. I. Tayasu I. Abe T. (2000). Role of the mutualistic fungus in lignin degradation in the fungus-growing termite *Macrotermes gilvus* (*Isoptera*; *Macrotermitinae*). *Soil Biol. Biochem.* 32 653–658. 10.1016/S0038-0717(99)00192-3

[B24] Kirk P. M. Cannon P. F. Minter D. W. Stalpers J. A. (2008). *Dictionary of the Fungi*, 10th Edn Wallingford: CABI.

[B25] Koljalg U. Nilsson R. Abarenkov K. Tedersoo L. Taylor Andy F. S. Bahram M. (2013). Towards a unified paradigm for sequence-based identification of fungi. *Mol. Ecol.* 22 5271–5277. 10.1111/mec.12481 24112409

[B26] Koutrotsios G. Danezis G. Georgiou C. Zervakis G. I. (2020). Elemental content in *Pleurotus ostreatus* and *Cyclocybe cylindracea* mushrooms: correlations with concentrations in cultivation substrates and effects on the production process. *Molecules* 25:2179. 10.3390/molecules25092179 32392710 PMC7249068

[B27] Lee C. Y. Park J. E. Kim B. B. Kim S. M. Ro H. S. (2009). Determination of mineral components in the cultivation substrates of edible mushrooms and their uptake into fruiting bodies. *Mycobiology* 37 109–113. 10.4489/MYCO.2009.37.2.109 23983518 PMC3749399

[B28] Li H.-J. Dietrich C. Zhu N. Mikaelyan A. Ma B. Pi R. (2016). Age polyethism drives community structure of the bacterial gut microbiota in the fungus-cultivating termite *Odontotermes formosanus*. *Environ. Microbiol.* 18 1440–1451. 10.1111/1462-2920.13046 26346907

[B29] Li H.-J. Sun J.-Z. Zhao J.-M. Deng T.-F. Lu J.-R. Dong Y. (2012). Physicochemical conditions and metal ion profiles in the gut of the fungus-growing termite *Odontotermes formosanus*. *J. Insect Physiol.* 58 1368–1375. 10.1016/j.jinsphys.2012.07.012 22858833

[B30] Li H.-J. Yelle D. J. Li C. Yang M.-Y. Ke J. Zhang R.-J. (2017). Lignocellulose pretreatment in a fungus-cultivating termite. *PNAS* 114 4709–4714. 28424249 10.1073/pnas.1618360114 PMC5422824

[B31] Liang S.-Y. Wang C.-P. Ahmad F. Yin X.-J. Hu Y. Mo J.-C. (2020). Exploring the effect of plant substrates on bacterial community structure in termite fungus-combs. *PLoS One* 15:e0232329. 10.1371/journal.pone.0232329 PMC7194444 32357167

[B32] Makonde H. M. Boga H. I. Osiemo Z. Mwirichia R. Stielow J. B. Goker M. (2013). Diversity of *Termitomyces* associated with fungus-farming termites assessed by cultural and culture-independent methods. *PLoS One* 8:e56464. 10.1371/journal.pone.0056464 23437139 PMC3577893

[B33] Makonde H. M. Mwirichia R. Muwawa E. M. Klenk H. P. Boga H. I. (2017). Fungal diversity and community structure in gut, mound and surrounding soil of fungus-cultivating termites. *Afr. J. Microbiol. Res.* 11 504–515. 10.5897/AJMR2017.8484

[B34] Makonde H. M. Mwirichia R. Osiemo Z. Boga H. I. Klenk H. P. (2015). 454 Pyrosequencing-based assessment of bacterial diversity and community structure in termite guts, mounds and surrounding soils. *Springerplus* 4:471. 10.1186/s40064-015-1262-6 26355944 PMC4556716

[B35] Mueller U. G. Gerardo N. Aanen D. K. Six D. L. Schultz T. R. (2005). The evolution of agriculture in insects. *Annu. Rev. Ecol. Evol. Syst.* 36 563–595. 10.1146/annurev.ecolsys.36.102003.152626

[B36] Odelson D. A. Breznak J. A. (1983). Volatile fatty acid production by the hindgut microbiota of xylophagous termites. *Appl. Environ. Microbiol.* 45 1602–1613. 16346296 10.1128/aem.45.5.1602-1613.1983 PMC242507

[B37] Ono K. Hata T. Yoshimura T. Kinjo K. (2016). Wood decaying properties of the termite mushroom *Termitomyces eurrhizus*. *J. Wood Sci.* 63 83–94. 10.1007/s10086-016-1588-x

[B38] Otani S. Mikaelyan A. Nobre T. Hansen L. H. Kone N. A. Sorensen S. J. (2014). Identifying the core microbial community in the gut of fungus-growing termites. *Mol. Ecol.* 23 4631–4644. 10.1111/mec.12874 25066007

[B39] Pielou E. C. (1966). The measurement of diversity in different types of biological collections. *J. Theor. Biol.* 13 131–144. 10.1016/0022-5193(66)90013-0

[B40] Poulsen M. Hu H. Li C. Chen Z. Xu L. Otani S. (2014). Complementary symbiont contributions to plant decomposition in a fungus-farming termite. *PNAS* 111 14500–14505. 10.5524/100055 25246537 PMC4209977

[B41] Quast C. Pruesse E. Yilmaz P. Gerken J. Schweer T. Yarza P. (2013). The SILVA ribosomal RNA gene database project: improved data processing and web-based tools. *Nucleic Acids Res.* 41 D590–D596. 10.1093/nar/gks1219 23193283 PMC3531112

[B42] Sawhasan P. Worapong J. Flegel T. W. Vinijsanun T. (2012). Fungal partnerships stimulate growth of *Termitomyces clypeatus* stalk mycelium in vitro. *World J. Microbiol. Biotechnol.* 28 2311–2318. 10.1007/s11274-012-1038-x 22806105

[B43] Shannon C. E. (1948). A mathematical theory of communication. *Bell System Tech. J.* 27 379–423, 623–656. 10.1002/j.1538-7305.1948.tb00917.x

[B44] Shik J. Z. Gormez E. Kooij P. W. Santos J. C. Wcislo W. T. Boomsma J. J. (2016). Nutrition mediates the expression of cultivar-farmer conflict in a fungus-growing ant. *PNAS* 113 10121–10127. 27551065 10.1073/pnas.1606128113 PMC5018747

[B45] Shinzato N. Muramatsu M. Watanabe Y. Matsui T. (2005). Termite-regulated fungal monoculture in fungus combs of a macrotermitine termite *Odontotermes formosanus*. *Zool. Sci.* 22 917–922. 10.2108/zsj.22.917 16141705

[B46] Sidde Gowda D. K. Rajagopal D. (1990). Association of *Termitomyces* spp. with fungus growing termites. *Proc. Indian Acad. Sci.* 99 311–315. 10.1007/BF03186400

[B47] Siddiquee S. Rovina K. Naher L. Rodrigues K. F. Uzzaman A. Z. (2015). Phylogenetic relationships of *Termitomyces aurantiacus* inferred from internal transcribed spacers DNA sequences. *Adv. Biosci. Biotechnol.* 06 358–367. 10.4236/abb.2015.65035

[B48] Simpson E. H. (1949). Measurement of diversity. *Nature* 163:688 10.1038/163688a0

[B49] Steinberg R. A. (1950). Growth of fungi in synthetic nutrient solutions. *Bot. Rev.* 16 208–228. 10.1007/bf02878505

[B50] Stewart W. D. P. (1980). Some aspects of structure and function in N2-fixing cyanobacteria. *Ann. Rev. Microbiol.* 34 497–536. 10.1146/annurev.mi.34.100180.002433 6108091

[B51] Thorburn A. N. Macia L. Mackay C. R. (2014). Diet, metabolites, and “western-lifestyle” inflammatory diseases. *Immunity* 40 833–842. 10.1016/j.immuni.2014.05.014 24950203

[B52] Tibuhwa D. D. (2012). *Termitomyces* species from Tanzania, their cultural properties and unequalled basidiospores. *JBLS* 3 140–159. 10.4172/2157-7463.1000117

[B53] Toyama-Kato Y. Yoshida K. Fujimori E. Haraguchi H. Shimizu Y. Kondo T. (2003). Analysis of metal elements of hydrangea sepals at various growing stages by ICP-AES. *Biochem. Eng. J.* 14 237–241. 10.1016/S1369-703X(02)00220-6

[B54] Visser A. A. Nobre T. Currie C. R. Aanen D. K. Poulsen M. (2012). Exploring the potential for actinobacteria as defensive symbionts in fungus-growing termites. *Microb. Ecol.* 63 975–985. 10.1007/s00248-011-9987-4 22173371

[B55] Whitaker A. (1976). Amino acid transport into fungi: an essay. *Trans. Br. Mycol. Soc.* 67 365–376. 10.1016/S0007-1536(76)80160-X

[B56] Wu G. (2009). Amino acids: metabolism, functions, and nutrition. *Amino Acids* 37 1–17. 10.1007/s00726-009-0269-0 19301095

[B57] Yang G.-Y. Ahmad F. Liang S.-Y. Fouad H. Guo M.-X. Gaal H. A. (2020). *Termitomyces heimii* associated with fungus-growing termite produces Volatile Organic Compounds (VOCs) and lignocellulose-degrading enzymes. *Appl. Biochem. Biotechnol.* 192 1270–1283. 10.1007/s12010-020-03376-w 32720080

[B58] Yin C. Jin L. Li S. Xu X. Zhang Y. (2019). Diversity and antagonistic potential of Actinobacteria from the fungus-growing termite *Odontotermes formosanus*. *3 Biotech.* 9:45. 10.1007/s13205-019-1573-3 30729069 PMC6342738

[B59] Zeleke J. Gessesse A. Abate D. (2013). Substrate-utilization properties of *Termitomyces* culture isolated from termite mound in the great rift valley region of Ethiopia. *J. Nat. Sci. Res.* 3 16–21.

[B60] Zhao G. Nyman M. Jonsson J. A. (2006). Rapid determination of short-chain fatty acids in colonic contents and faeces of humans and rats by acidified water-extraction and direct-injection gas chromatography. *Biomed. Chromatogr.* 20 674–682. 10.1002/bmc.580 16206138

[B61] Zhao H.-J. Li S.-S. Zhang J.-J. Che G. Zhou M. Liu M. (2016). The antihyperlipidemic activities of enzymatic and acidic intracellular polysaccharides by *Termitomyces albuminosus*. *Carbohydr. Polym.* 151 1227–1234. 10.1016/j.carbpol.2016.06.058 27474674

