# Supplementary material for: Investigation of Physicochemical Indices and Microbial Communities in Termite Fungus-Combs
Source: Front Microbiol. 2021 Jan 15;11:581219. doi: 10.3389/fmicb.2020.581219 (PMC7843810; doi:10.3389/fmicb.2020.581219)
Supplement: Supplementary file 1 [file Data_Sheet_1.pdf]

Supplementary Material

Supplementary Figures and Tables

Abbreviations

CF: Combs with *Termitomyces* basidiomes

CNF: Combs without *Termitomyces* basidiomes

HPLC: High Performance Liquid Chromatography

ICP-OES: Inductively Coupled Plasma Optical Emission Spectrometer

GC-FID: Gas Chromatography Equipped with a Flame Ionization Detector

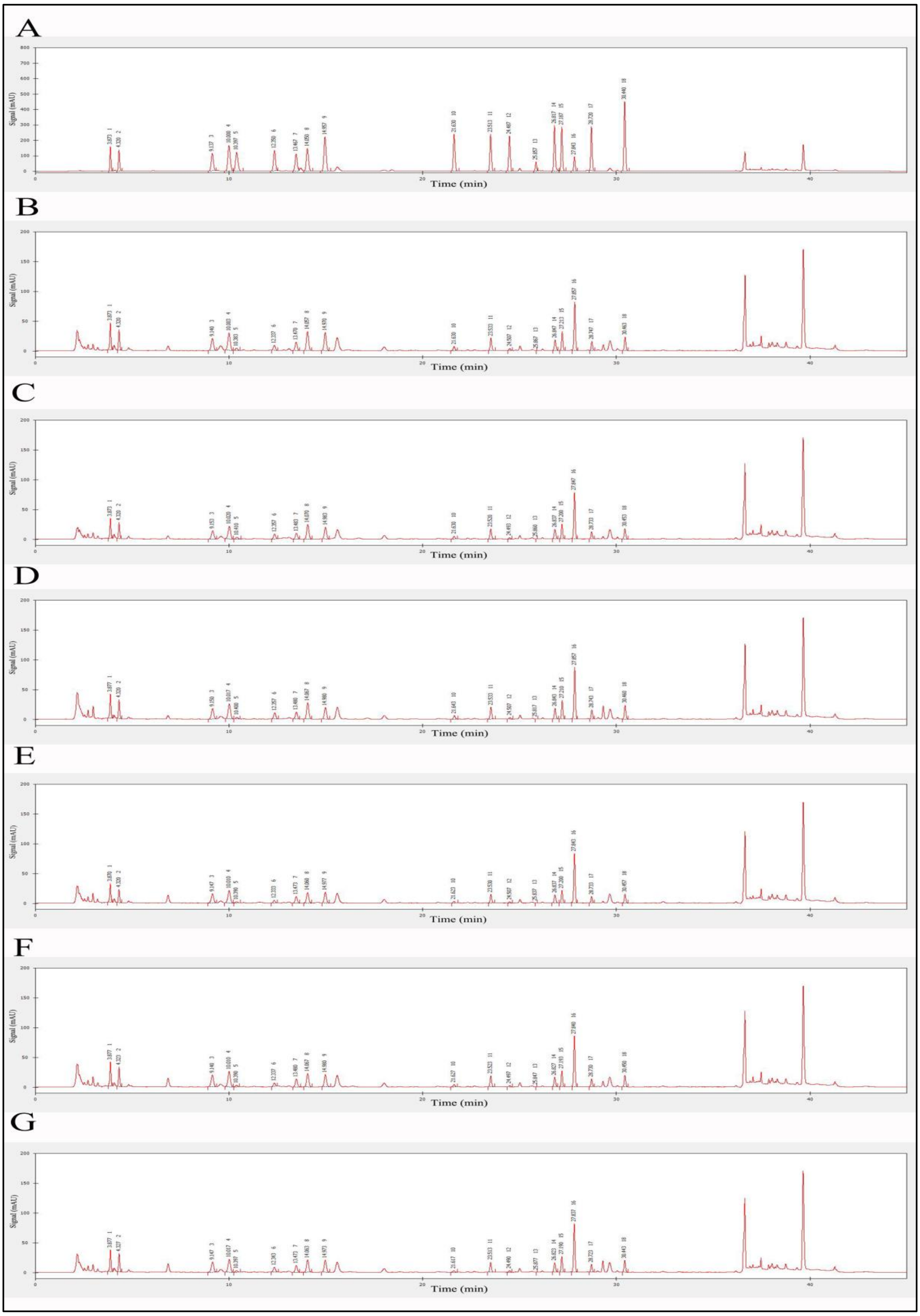

Supplementary Figure 1. Chromatograms of different amino acids in standard solution and six combs (A in CNF1, B in CNF2, C in CNF3, D in CF1, E in CF2, F in CF3 and G in standard solutions). Different peaks represent various amino acids (1=Asp, 2=Glu, 3=Ser, 4=Gly, 5=His, 6=Arg, 7=Thr, 8=Ala, 9=Pro, 10=Tyr, 11=Val, 12=Met, 13=Cys, 14=Ile, 15=Leu, 16=Phe, 17=Lysine).

Table S1. Parameters of ICP-OES

RF power	1150 W
Nebulizer gas flow rate	General speed
Auxiliary gas flow rate	0.5 L/min
Nebulizer gas flow rate	General speed
Read	Peak area
Read delay	5.0 s
Replicates	3
Purge gas	Nitrogen
Shear gas	Air
Plasma gas	Argon

Table S2. Comparative analysis of seventeen amino acids in CNF and CF

Amino acids	CNF(μg/g)	CF(μg/g)	Significance
Aspartic acid	2107.24 ±169.44	1910.73 ±109.67	0.394
Glutamic acid	1864.38 ±104.48	1708.38 ±175.92	0.497
Serine	1004.41 ±85.94	1030.54 ±65.32	0.821
Glycine	668.49 ±53.19	593.10 ±23.37	0.293
Histidne	224.69 ±19.32	158.05 ±11.93	0.053
Arginine	703.53 ±49.87	473.08 ±106.83	0.151
Threonine	711.03 ±73.31	709.07 ±36.17	0.982
Alanine	984.09 ±69.92	703.08 ±41.88	0.036
Proline	687.73 ±106.31	577.65 ±30.66	0.412
Tyrosine	270.80 ±22.43	147.46 ±14.27	0.014
Valine	558.32 ±21.72	463.94 ±29.83	0.069
Methionine	105.95 ±18.56	88.99 ±9.67	0.477
Cysteine	5.70 ±1.93	6.05 ±1.63	0.895
Isoleucine	480.46 ±7.14	411.82 ±12.61	0.016
Leucine	763.47 ±47.58	633.96 ±46.32	0.123
Phenylalanine	468.37 ±26.69	399.84 ±29.27	0.159
Lysine	393.37 ±25.32	332.99 ±29.24	0.195

Table S3. Linearity correlations of fatty acids in combs

Fatty acids	Regression equation	r^2
Acetic acid	y = 1.6694x + 16.980	0.9987
Propionic acid	y = 1.0827x + 15.598	0.9987
n-Butyric acid	y = 0.8749x + 20.507	0.9969
n-Valeric acid	y = 0.7994x + 25.341	0.9949
n-Caproic acid	y = 0.7737x + 28.552	0.9925
n-Heptanoic acid	y = 0.7536x + 33.071	0.9878

x = slope; y = intercept; r= correlation coefficient.

Table S4. Average contents of different fatty acids in CNF and CF combs.

Combs	Formic acid(μg/g)	Acetic acid(μg/g)	Propionic acid(μg/g)	n-Butyric acid(μg/g)	n-Valeric acid(μg/g)	n-Caproic acid(μg/g)	n-Heptanoic acid(μg/g)
CNF	NF	47.89±1.21	1.09±0.01	57.80±0.92	1.37±0.02	64.54±1.20	1.73±0.05
CF	NF	53.86±4.11	1.00±0.04	74.78±7.93	1.20±0.12	94.59±11.39	1.63±0.22
Sig	/	0.28	0.18	0.16	0.30	0.06	0.68

NF means not found.

Table S5. Numbers of sequences, coverage, richness, diversity, and evenness indicators in combs.

Combs	Number of Sequences	Coverage (%)	Shannon	Simpson	Chao1	Observed species	Pielou's evenness
CNF1	21566	99.60	0.59	0.89	469.56	413.50	0.59
CNF2	22554	99.62	0.72	0.96	625.81	579.20	0.72
CNF3	22536	99.64	0.72	0.97	512.44	465.30	0.72
CF1	24198	99.65	0.59	0.90	489.10	443.30	0.59
CF2	23618	99.63	0.57	0.90	392.41	292.70	0.57
CF3	23042	99.72	0.61	0.90	424.38	385.70	0.61

Table S6. Comparisons of bacterial community in CF and CNF at phylum level.

Phylum	CNF (%)				CF (%)				Sig
	CNF1	CNF2	CNF3	CNF average	CF1	CF2	CF3	CF average	
Proteobacteria	68.52	23.97	36.75	43.08	61.46	58.90	55.77	58.71	0.359
Bacteroidetes	18.85	43.83	37.40	33.36	19.25	20.90	19.36	19.84	0.146
Firmicutes	5.00	16.44	15.18	12.21	5.65	3.02	2.45	3.71	0.135
Actinobacteria	1.63	2.58	2.02	2.08	4.28	4.58	6.74	5.20	0.044
Cyanobacteria	1.46	0.85	1.55	1.29	2.03	2.92	2.84	2.59	0.024
Acidobacteria	0.16	0.20	0.10	0.16	0.91	0.15	2.08	1.05	0.252
Spirochaetes	0.35	2.50	0.52	1.12	0.24	0.03	0.00	0.09	0.211
Synergistetes	0.09	2.54	0.37	1.00	0.00	0.00	0.00	0.00	0.266
Planctomycetes	0.55	0.94	0.67	0.72	0.22	0.04	0.02	0.09	0.016
Gemmatimonadetes	0.13	0.00	0.00	0.04	0.13	0.02	2.11	0.75	0.356
Chloroflexi	0.17	0.05	0.06	0.09	0.39	0.02	1.24	0.55	0.333
Candidate_phylum_TM7	0.24	0.58	0.28	0.37	0.12	0.00	0.36	0.16	0.242
Chlorobi	0.29	0.62	0.20	0.37	0.00	0.00	0.00	0.00	0.044
Armatimonadetes	0.07	0.16	0.14	0.13	0.02	0.32	0.16	0.17	0.673
Candidate_phylum_TM6	0.03	0.00	0.02	0.02	0.55	0.00	0.02	0.19	0.391
Candidate_phylum_TG3	0.02	0.19	0.41	0.20	0.00	0.00	0.00	0.00	0.209
Elusimicrobia	0.00	0.21	0.19	0.13	0.00	0.06	0.00	0.02	0.227
Nitrospirae	0.00	0.00	0.05	0.02	0.02	0.22	0.12	0.12	0.210
Deferribacteres	0.00	0.04	0.24	0.09	0.00	0.00	0.00	0.00	0.277
Candidate_phylum_WS3	0.00	0.00	0.00	0.00	0.00	0.00	0.27	0.09	0.374

Table S7. Bacterial community structure in CF and CNF at family level.

Family	CNF (%)				CF (%)				Sig
	CNF1	CNF2	CNF3	CNF average	CF1	CF2	CF3	CF average	
Brucellaceae	7.82	8.04	9.90	8.59	13.27	20.80	23.56	19.21	0.069
Chitinophagaceae	8.20	9.16	9.14	8.83	16.52	18.85	18.21	17.86	0.002
Rikenellaceae	6.25	25.84	17.64	16.58	0.72	1.25	0.02	0.66	0.107
Burkholderiaceae_1	11.72	1.96	6.92	6.87	21.96	4.44	0.06	8.82	0.807
Bradyrhizobiaceae	29.78	0.89	0.71	10.46	1.64	5.90	1.34	2.96	0.486
Thermaceae	2.35	3.76	3.74	3.28	3.94	8.53	5.96	6.14	0.154
Comamonadaceae	1.64	2.70	3.02	2.45	4.51	5.22	5.37	5.04	0.010
Lachnospiraceae	3.12	6.43	5.79	5.11	2.94	0.93	0.76	1.54	0.051
Rhizobiaceae	1.94	1.79	1.52	1.75	3.72	5.31	3.91	4.31	0.030
Caulobacteraceae	1.29	1.19	2.25	1.58	2.56	3.17	3.16	2.97	0.034
Moraxellaceae	3.30	0.87	1.05	1.74	1.38	2.03	1.37	1.59	0.871
Sphingomonadaceae	1.54	0.82	2.48	1.61	0.97	1.84	2.29	1.70	0.895
Ruminococcaceae	0.67	5.03	2.24	2.65	0.70	0.22	0.44	0.45	0.226
Porphyromonadaceae_1	0.20	2.26	5.09	2.52	0.21	0.05	0.00	0.09	0.228
Pseudomonadaceae	2.46	0.19	0.60	1.08	0.84	0.84	1.56	1.08	0.997
Methylobacteriaceae	0.58	0.51	0.65	0.58	0.94	1.19	2.21	1.45	0.091
Enterobacteriaceae	1.94	0.29	1.25	1.16	0.33	0.87	0.88	0.69	0.439
Porphyromonadaceae_3	0.58	2.38	1.74	1.57	0.09	0.10	0.00	0.06	0.103
Phyllobacteriaceae	0.48	0.40	0.63	0.50	0.82	1.29	1.17	1.10	0.035
Burkholderiaceae_2	0.36	0.49	0.46	0.43	1.45	0.87	1.00	1.11	0.056

Table S8. Bacterial diversity comparisons in CF and CNF at genus level.

Genus	CNF (%)				CF (%)				Sig
	CNF1	CNF2	CNF3	CNF average	CF1	CF2	CF3	CF average	
Sediminibacterium	8.18	9.15	9.14	8.82	16.51	18.80	18.19	17.83	0.002
Alistipes_II	4.32	18.99	11.31	11.54	0.42	0.98	0.02	0.47	0.120
Burkholderia_1	3.85	0.08	0.15	1.36	21.40	4.36	0.01	8.59	0.384
Thermus	2.35	3.76	3.74	3.28	3.94	8.53	5.96	6.14	0.154
Pelomonas	1.25	2.29	2.43	1.99	3.21	3.97	2.76	3.31	0.062
Alisitpes_IV	1.22	4.18	3.74	3.05	0.29	0.23	0.00	0.17	0.036
Acinetobacter	2.57	0.78	0.89	1.41	1.21	1.93	1.34	1.49	0.907
Sphingomonas_3	1.34	0.69	2.41	1.48	0.93	1.58	1.73	1.41	0.913
Dysgonomonas	0.20	2.26	5.09	2.52	0.21	0.05	0.00	0.09	0.228
Brevundimonas	0.68	0.52	1.23	0.81	1.62	1.60	1.70	1.64	0.019
Methylobacterium	0.58	0.51	0.65	0.58	0.87	1.14	2.21	1.41	0.115
Candidatus_Arthromitus	0.59	2.66	0.93	1.39	1.02	0.05	0.02	0.36	0.249
Tannerella	0.58	2.31	1.74	1.54	0.09	0.10	0.00	0.06	0.100
Amycolatopsis	0.27	0.40	0.15	0.27	0.52	1.31	0.79	0.87	0.112
Cupriavidus	0.26	0.33	0.32	0.30	1.09	0.29	0.89	0.76	0.134
Pseudomonas_1	0.21	0.13	0.26	0.20	0.45	0.64	1.16	0.75	0.118
Treponema_Ia	0.20	1.87	0.45	0.84	0.23	0.03	0.00	0.08	0.225
Novispirillum	0.13	0.09	0.57	0.27	0.40	0.65	0.84	0.63	0.143
Brevibacterium	0.32	0.08	0.28	0.23	0.48	0.63	0.60	0.57	0.024
Bacteroides	0.36	1.19	0.72	0.76	0.10	0.06	0.00	0.05	0.098

Table S9. Numbers of sequences, coverage and five alpha diversity indexes for every comb.

Combs		Number of Sequences	Coverage (%)	Shannon	Simpon	Chao1	Observed species	Pielou's evenness
CNF	CNF1	4032	100.00	1.87	0.671	8	8	0.62
	CNF2	4992	100.00	0.01	0.002	2	2	0.01
	CNF3	4324	100.00	0.18	0.052	2	2	0.18
CF	CF1	5854	99.99	0.01	0.002	2	2	0.01
	CF2	4008	100.00	1.02	0.416	7	7	0.36
	CF3	4910	99.99	0.75	0.328	4	4	0.37
Significance		/	/	0.83	0.83	0.82	0.82	0.83
